# Foreign Summary

**Published:** 1838-07-18

**Authors:** 


### FOREIGN SUMMARY.

*Instrument for the dilatation of the Meatus Urinarius.*—The obstacle, opposed by a narrow meatus urinarius, particularly in children, to the operation of lithotrity, Dr. Labat, of Paris, proposes to remedy by the following dilating instrument. It is shaped like a small key, having, in place of wards, two blades, eight or ten lines in length, which, when brought together, form a stem, slightly conoidal, and fine enough to enter the narrowest meatus. Each of these blades, being traversed at its base, by a quadrilateral groove and screw, they may be gradually separated, so as to give the meatus a dilatation, sufficient to receive lithotrity instruments of the largest size. This instrument the doctor has also successfully used for the dilatation of a stricture, situated five lines from the meatus urinarius.—*Gazette des Hopitaux.*

---

*Results of the Revaccinations, practised in the Prussian Army, during the year* 1836.—42,124 individuals were revaccinated, 32,633 of whom bore the cicatrices of previous vaccination. In 6,545, these cicatrices were doubtful; in 2,840, there were none. In the revaccinations of this year, there were regular pustules in 18,136 individuals, irregular in 9,940. In 14,048, vaccination was at first unsuccessful, but, upon repetition, it succeeded in 1,569, and failed again in 8,205 cases. The number of pustules varied in the different individuals from 1 to 30. Among those who had regular pustules, 7,311 had from 1 to 5 pustules; 5,647 from 6 to 10; 4,418 from 10 to 20, and 700 from 21 to 30.

Of the individuals, revaccinated in 1836 or previously, 14 were during this year affected with varicella, and 8 with varioloid. But there was not a single case of pure variola.

The vaccination was in general practised with vaccine matter, freshly taken from the arms of infants, although it was sometimes necessary to use that taken from adults. Stale virus was rarely employed. The surgeons of the different corps disagreed in opinion, as to the greater or less efficacy of the virus from children or from adults; but direct experience would seem to show very little difference in their capacity of producing pustules.

If the results obtained in 1836, are compared with those of preceding years, the conclusion is arrived at, that the susceptibility of contracting a new vaccination and consequently variola, increases every year. Of 42,124 individuals revaccinated in 1836, 18,136 cases were successful. In 1833, 48,478 persons revaccinated furnished only 15,269 successful cases; in 1834, of 44,454 revaccinations, 16,679 were successful; and in 1835, of 39,192, 15,315 were successful. The successful cases were, therefore, to the whole number, in the proportion of 31 per cent. in 1833, of 37 in 1834, of 39 in 1835, and of 43 in 1836.

In 1833, a first revaccination was unsuccessful in 4,161 subjects; but, repeated, it succeeded in 784; of 6,830 cases not followed by success in 1834, the repetition of the operation succeeded in 866; in 1835, in 9,411, it succeeded 1,465 times; and, in 1836, 1,569 times in 9,744 individuals.—*Medicin. Zeitung.*

---

*On the Foreign Bodies which serve as Nuclei for Urinary Calculi, by M. Civiale.*—At a late meeting of the Academy of Sciences, M. Civiale made some observations on certain modes of formation of calculi, an abstract of which cannot fail to be interesting.

An account of the nuclei which occasionally serve for the formation of urinary calculi is a neglected, though curious, point in the history of the latter productions. M. Civiale has made some researches with the view of clearing up several obscure facts, and has assembled together 166 cases, from an analysis of which it appears that the nucleus of the stone was formed in 32 cases by needles or pins; in 21 by bougies or catheters; in 14 by pieces of wood; in 13 by bullets; in 24 by fragments of bones, pipe stoppers, barometer tubes, or stems of plants; in 14 by the beards of barley or hairs; in 4 by pledgets of lint, and finally by rings, nails, fruit-stones, needle-cases, &c.

The greater part of the above cases present several interesting points, but we must confine our notice to those of recent date.

Pins or needles are the foreign bodies which most frequently serve as nuclei for stone. This probably depends on the facility with which they present themselves to a certain class of depraved individuals, although it is difficult to conceive in what manner females, more especially, can satisfy their passions with such instruments. A few needles, of from five to six inches in length, remained a considerable time in the bladder without having their points covered with calculous matter, and in a few cases only did they penetrate the bladder, vagina, perineum, &c.

The formation of calculi upon bullets, and other foreign bodies of a similar nature, gives rise to several physiological considerations of importance. In many cases these bodies have passed into the bladder through passages which the surgeon would never dare to traverse with the knife, and what is more remarkable, without producing, in many cases, any notable disorder or inconvenience. Thus, for example, pieces of wood from three to seven inches in length, bullets, &c., have remained for years in the bladder without giving rise to the insupportable pain, necessity of frequent micturition, &c., which usually attend the presence of foreign bodies in that viscus.

The introduction of the nucleus is either accidental or intentional. In the former case the foreign body has found its way into the bladder in consequence of a wound, of a fall, of some fistulous communication between the intestine and bladder, or finally through the fault of the surgeon; in the latter case it, generally speaking, has been introduced by the patient himself, sometimes for the purpose of relieving a retention of urine, or pushing back a calculus; sometimes in a fit of momentary derangement; but most frequently in consequence of depraved and lascivious ideas. The effects produced by the foreign bodies in the bladder are extremely various. Sometimes they generate the highest degree of suffering and pain, which terminate rapidly in death; in other cases they seem to be scarcely felt by the organ, or the inconvenience which they occasion is purposely concealed by the sufferer.

In a therapeutical point of view the presence of these foreign bodies in the bladder is a matter of much interest. From the table to which we have already alluded, it would appear that in 12 cases only, of the 166, they were discharged spontaneously either from the bladder or by an artificial passage. This is a curious circumstance, when we consider that in many cases the bodies themselves are small, and of a rounded form. In 64 cases the operation of lithotomy was performed; the difficulty of the operation depending much on the size and shape of the nucleus of the stone. In 26 cases the foreign bodies were extracted through the urethra, without the aid of a cutting instrument. The majority of such facts is recent and connected with lithotrity. M. Civiale has already published six cases in which he has extracted with success two elastic bougies, a bean, a pea, a stem of a plant, and a piece of straw. In his present communication he details two more cases; in one of which he extracted a fragment of a waxen bougie; in another, a portion of a barometer tube about three inches in length.—*London Lancet, from French Gazette*, April, 1838.

---

*Mode of obtaining Creosote.*—The following is an economical method of obtaining creosote, proposed by M. Cozzi. A quantity of tar is distilled in an alembic, and the products collected in a cylindrical vessel half filled with water. The products are acetic acid, eussion, parafin, and creosote, which latter is recognised by its specific gravity. The impure creosote is isolated from the other products by means of a syphon, and on this being done, sulphuric acid, weakened with one-half water, is added; the creosote now mounts to the surface of this fluid, which is warmed by an admixture of boiling dilute sulphuric acid, and the supernatant fluid is drawn off and placed in an open-mouthed bottle, one-third filled. This is exposed to the air for three days, and the product is again distilled, when a reddish fluid is obtained. The latter having been treated thrice in a similar manner, furnished pure creosote, limped as water, of 1.007 specific gravity, and boiling at 205° R.—*Ib., from Jour. de Chem. Med.*, May, 1838.

